# Acknowledgement to Reviewers of *Pharmaceuticals* in 2018

**DOI:** 10.3390/ph12010010

**Published:** 2019-01-09

**Authors:** Pharmaceuticals Editorial Office

**Affiliations:** MDPI, St. Alban-Anlage 66, 4052 Basel, Switzerland

Rigorous peer-review is the corner-stone of high-quality academic publishing. The editorial team greatly appreciates the reviewers who contributed their knowledge and expertise to the journal’s editorial process over the past 12 months. In 2018, a total of 135 papers were published in the journal, with a median time to first decision of 16 days and a median time to publication of 37 days. The editors would like to express their sincere gratitude to the following reviewers for their cooperation and dedication in 2018:

Ábrányi-Balogh, PéterLee, HowardAdam, MikeLee, ZhenghongAdams, JamesLeggat, PeterAdriana, Estrada-BernalLehmann, ChristianAkopian, ArmenLeppert, WojciechAl Massadi, OmarLi, Xiang-GuoAlbano, EmanueleLi, YvonneAldini, GiancarloLiang, YongAl-Horani, RamiLin, Hsiao-YiAllinquant, BernadetteLin, Kuo-ShyanAntal, IstvánLin, Liang-TzungAnticoli, SimonaLinder, MariaAntoni, GunnarLinial, Maxine L.Arano, YasushiLitchfield, DavidAviles, Francesc XavierLiu, Hai-yangBaek, Jong-SuepLiu, YuBaliraine, FrederickLiu, ZhichaoBansal, AdityaLobo-Castañón, María JesúsBarbu, MagdaLobocka, MalgorzataBernini, RobertaLocatelli, MarcelloBesson, ThierryLoc-Carrillo, CatherineBiagini, GiuseppeLonardo, AmedeoBiagini, GiuseppeLopez Rodilla, Jesus M.Bier, DirkLundborg, MagnusBiosca, Elena G.Luxen, AndréBorup Jensen, SvendMalik, DanishBozzi, YuriMalinowska, BarbaraBratt, AlisonMancias, Joseph D.Brudzynski, KatrinaManconi, MariaBrust, PeterMano, YujiCabantchik, IoavMarcello, ElenaCafiero, MauricioMarchant, Jonathan S.Cametti, CesareMarco-Contelles, JoséCardinale, JensMartins, Ana CatarinaCardoso, SusanaMaruyama, KeiCarlos, Ana RitaMarx, Joannes J. M.Casu, CarlaMásson, MárCatalano, AntoninoMassotte, DominiqueCerón-Carrasco, José PedroMatthews, SusanChami, BelalMattii, LetiziaChanishvili, NinaMcKeague, MaureenChappell, JosephMedina, DiegoChen, DaiqinMeldolesi, JacopoChen, HongweiMelman, ArtemChen, Po-YuanMergler, StefanChiabrando, DeborahMerlini, LucioChiarelli, LaurentMietlicki-Baase, ElizabethChin, PaulMompar, lerRichardChou, Kuo-ChenMontenegro, LuciaChubanov, VladimirMontesarchio, DanielaChudasama, VijayMortiboys, HeatherChung, Man-KyoMuccioli, GiulioCichero, ElenaMurray, Thomas ScotCirillo, GiuseppeNaftalin, RichardCollins, ColmNairz, ManfredConti, BiceNanjundan, MeeraCorchado, Jose C.Nataf, SergeCortés, Maria PilarNikolakakis, IoannisCostantino, LucaNitulescu, George MihaiCraig, JeffreyNørskov-Lauritsen, NielsCrisponi, GuidoNtie-Kang, FideleCsiszár, AgnesOcampo-Garcia, BlancaDal Ben, DiegoOh, Seung HyunDanilenko, MichaelOkamoto, ShigefumiDas, AditiOkino, TatsufumiDaugelavicius, RimantasOnyango, IsaacDe Franciscis, VittorioOrman, MehmetDebourdeau, P.Pabbidi, Reddy MDemopoulos, Vassilis J.Padrón, José M.Denny, BillPantopoulos, KostasDeriu, Marco AgostinoPasut, GianfrancoDeRosa, MariaPellegrino, SaraD'hooge, Dagmar R.Perez-Stable, CarlosDi Fazio, PietroPeterson, Matt A.Di Marino, DanielePietzsch, Hans-JürgenDi Martino, PieraPilc, AndrzejDiamond, GillPinna, LorenzoDíez, DavidPissarek, MargitDoi, HisashiPlanas, MartaDouroumis, DennisPopa-Wagner, AurelDubos, ChristianPorter, ToddDuburs, GunarsPoudel, BarunDunaief, JoshuaPoznański, JarosławEl Aidy, SaharQuadir, MohiuddinElder, JohnRahimi, FaridEllenbroek, BartRamachandran, AnupElsheikha, Hany MRamadori, GiulianoEndoh, TamakiRescifina, AntonioEppard, ElisabethRistori, SandraEritja, RamonRivera, VictorEspuny, Antoni CaminsRodolfo, CarloFairlamb, AlanRodriguez, Marcela GFalcão, AndreRoetto, AntonellaFernandes-Ferreira, ManuelRohacs, TiborFerris, WilliamRolan, PaulFiorio Pla, AlessandraRoselt, PeterFitzpatrick, LeoRosenthal, RitaFleig, AndreaRoubertoux, PierreFormisano, CarmenRovero, PaoloForsyth, Christopher B.Ruiz-Gómez, GloriaFoubelo, FranciscoSaiz, Juan-CarlosFranca, CodazziSantamaria, RitaFriman, Ville-PetriSantamaria, RitaFrischau, fIreneSantos-Filho, OsvaldoFujii, HideakiSawynok, JanaFurukawa, TatsuhikoSchreckenberger, MathiasGangloff, SophieSchubert, StephanieGeppetti, PierangeloScorziello, AntonellaGinsberg, StephenSemeraro, FabrizioGinzburg, Yelena ZSerpico, RosarioGomes, Maria S.Sgrignani, JacopoGowans, EricSheikh, M. SaeedGrant, Gregory A.Shen, Chwan-LiGreenwood, Michael T.Shen, YangGregáň, JurajShi, HuaGrimm, Marcus O. W.Shimizu, ShunichiGruber, ChristianSiciliano, CarloGuinamard, RomainSignore, GiovanniGuo, PeixuanSim, EdithGuttman, AndrasSingh, NeenaHaase, JanaSiniscalco, DarioHammer, lJens AndreSinko, PatrickHan, Byung WooSiracusa, ValentinaHans, GuySirdah, MahmoudHaqshenas, GholamrezaSlavik, RogerHardwick, James P.Sobkowski, MichalHay, Roderick J.Sonnet, PascalHingorani, DinaSt Hill, CatherineHoehenwarter, WolfgangStathopoulos, ConstantinosHollenstein, MarcelStephens, GaryHolsinger, DamianSturm, Brigitte N.Hong, HaoSurguchov, AndreiHope, SandraSvorc, LubomirHorvath, DragosSwenson, ErikHosohata, KeikoSzilágyi, AndrásHuang, Hui-ChiTaghibiglou, ChangizHuang, XudongTanner, Julian AlexanderHutter, MichaelTenson, TanelHwang, Youn-HwanTheodossiou, Theodossis AthanassiosIchikawa, HiroshiThèvenod, FrankImhof, PetraThompson, Miles D.Isidori, AlessandroTing, Richard C. H.Itarte, EmilioTitus, Mark A.Ivanov, AndreyTodeschini, RobertoIvens, Inge A.Tombelli, SaraIwano, HidetomoToyohara, JunIwatsuki, MasatoTrabace, LuigiaIzawa, TakeshiTrembley, JaneenJaiswal, Amit K.Tsakiris, Dimitrios A.Jaki, BirgitTseng, Chih-HuaJantas, DanutaTsourkas, PhilipposJeandet, PhilippeUberti, FrancescaJeney, ViktóriaVaidyanathan, GanesanJeong, Seon-YongVan Bennekom, W.P.Jimi, EijiroVan Den Bergh, HubertJohnson, R. JeremyVan Maarseveen, JanJones, MarjorieVázquez, M. EugenioJönsson, SivVenkatesan, JayachandranJules, KevinVergara, DanieleJulianna, OláhVerma, Navin KumarKaczor, Agnieszka A.Viola, GiampietroKadambi, Pradeep V.Vodnar, Dan CristianKafarski, PawelVolk, DavidKalhapure, RahulWadsak, WolfgangKalkman, Hans OWakao, MasahiroKanazawa, MasatoWall, Geoffrey C.Katapodis, PetrosWalters, Julian R. F.Kelly, Christopher V.Wang, XinwenKenna, Dervla T.D.Wei, HongpeiKevadiya, BhaveahWei, Zhao-JunKichkailo, AnnaWells, Richard A.Kiesewetter, Dale O.Wie, Myung-BokKim, Yun-baeWiese, SebastianKindrachuk, JasonWildemann, BrittKoivisto, AriWilliams, Christopher C.Kolodych, SergiiWong, Ching-OnKontoravdi, CleoWozniak-Knopp, GordanaKorabecny, JanWunderlich, GerdKotlinowski, JerzyYasuda, KazukiKrammer, FlorianYe, YanqiKumamoto, EiichiYoon, SorahKumar, DhirajYou, XiaonaKurokawa, MineoYoung, BryanLa Mendola, DiegoYuan, YaxiaLa Regina, GiuseppeZamyatnin, Jr.Andrey A.Lachman, JaromirZendo, TakeshiLartigue, LénaïcZhang, DeliangLatosińska, Jolanta NataliaZhang, WujieLatunde-Dada, Gladys OluyemisiZsombok, AndreaLawen, AlfonsZu, YouliLayek, BuddhadevZuvela, PetarLedo, Ana


